# Examining the influence of brain stimulation to the medial prefrontal cortex on the self‐reference effect in memory

**DOI:** 10.1002/brb3.2368

**Published:** 2021-11-03

**Authors:** Camill Burden, Ryan C. Leach, Allison M. Sklenar, Pauline Urban Levy, Andrea N. Frankenstein, Eric D. Leshikar

**Affiliations:** ^1^ University of Illinois at Chicago Chicago Illinois USA

**Keywords:** context memory, item memory, self‐reference memory effect, tDCS

## Abstract

Past work shows that processing information in relation to the self improves memory which is known as the self‐reference effect in memory. Other work suggests that transcranial direct current stimulation (tDCS) can also improve memory. Given recent research on self‐reference context memory effects (improved memory for contextual episodic details associated with self‐referential processing), we were interested in examining the extent stimulation might increase the magnitude of the self‐reference context memory effect. In this investigation, participants studied objects superimposed on different background scenes in either a self‐reference or other‐reference condition while receiving either active or sham stimulation to the dorsal medial prefrontal cortex (dmPFC), a cortical region known to support self‐reference context memory effects. Participants then completed a memory test that assessed item memory (have you seen this object before?) and context memory (with which background scene was this object paired?). Results showed a self‐reference context memory effect driven by enhanced memory for stimuli processed in the self‐reference compared to the other‐reference condition across all participants (regardless of stimulation condition). tDCS, however, had no effect on memory. Specifically, stimulation did not increase the magnitude of the self‐reference context memory effect under active compared to sham stimulation. These results suggest that stimulation of the dmPFC at encoding may not add to the memory benefits induced by self‐referential processing suggesting a boundary condition to tDCS effects on memory.

## Graphical abstract

In this investigation we examined the extent transcranial direct current (tDCS) applied to the dorsal medial prefrontal cortex (dmPFC) affects memory for information processed in reference to the self. We found strong evidence that self‐referential processing improved memory (relative to a control condition), but further showed that tDCS did not have strong effects on memory. Results advance understanding of the extent tDCS affects memory.

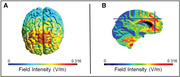

People develop a sense of self over a lifetime of experiences. This self‐schema influences many aspects of life including decision‐making, reasoning, and how experienced events are subsequently remembered (i.e., memory). Decades of research has shown that self‐relevant information tends to be better remembered than information that is not self‐relevant (Conway, 2005; Gutchess, Kensinger, Yoon, et al., 2007; Kesebir & Oishi, 2010; Markus, 1977; Rogers et al., 1977; Symons & Johnson, 1997). The reasoning behind the self‐reference effect is that self‐relevant information is easier to integrate into the existing schematic representation of the self and is thus more memorable (Conway, 2005; Conway & Pleydell‐Pearce, 2000), which follows from theoretical work suggesting that schematic representations have a strong effect on memory (Gilboa & Marlatte, 2017; Robin & Moscovitch, 2017; Van Kesteren et al., 2012). There have been many different procedures to examine the self‐reference effect in memory. Some experiments present words (such as the word, “kind”) and ask participants whether those words describe the self (Gutchess, Kensinger, & Schacter, 2007; Gutchess, Kensinger, Yoon, et al., 2007; Ilenikhena et al., 2021; Leshikar et al., 2015; Rogers et al., 1977). Other procedures have used less direct self‐reference manipulations by assessing participants’ personal preferences such as making subjective aesthetic judgments. For example, past studies have asked participants to decide whether they find stimuli such as pictures or images pleasant as a means to induce self‐referential processing (Dulas et al., 2011; Jacobsen et al., 2006; Leshikar & Duarte, 2012; Zysset et al., 2002). Across these different types of experimental procedures to induce self‐referential processing, participants make subjective, evaluative judgments about materials in line with their current schematic representations of the world (i.e., self‐schema).

The majority of experiments on the self‐reference effect in memory have used procedures to measure item memory (i.e., memory for individual items such as words or images; Symons & Johnson, 1997). More recent work, however, has examined self‐reference effects for context memory (Chen et al., 2016; Cunningham et al., 2014; Hamami et al., 2011; Rosa et al., 2016; Rosa & Gutchess, 2011; Serbun et al., 2011; Zhang et al., 2019). Such contextual details can include perceptual information (e.g., in which color was a word presented?), source details (e.g., was this spoken in a masculine or feminine voice?), or other materials simultaneously presented with an item (e.g., what picture was presented with this item?). Work investigating self‐reference context memory effects have typically found enhanced memory for context under self‐reference compared to control conditions. For example, in one investigation participants studied words presented with an array of contextual details (different fonts, different font colors, spoken by a masculine or feminine voice, etc.) under both self‐reference and semantic (control) processing conditions (Leshikar et al., 2015). Results showed improved memory for a variety of contextual details (perceptual, voice source) for words processed in the self‐reference relative to the semantic control condition. Such results, and others like it (Hamami et al., 2011; Serbun et al., 2011; Yin et al., 2019), suggest that processing information in reference to the self leads to detail‐rich memory representations. In this investigation, we examine the extent self‐reference context memory effects may be influenced by transcranial direct current stimulation (tDCS).

Research over the last decade has examined the extent tDCS influences memory (Brasil‐Neto, 2012; Coffman et al., 2014; Ferrucci et al., 2008; Friehs, Greene, et al., 2021; Hsu et al., 2015; Javadi & Cheng, 2013; Javadi et al., 2012; Javadi & Walsh, 2012; Leach et al., 2019; Manenti et al., 2013; Matzen et al., 2015). tDCS works by passing a mild electrical current through the brain that induces electric fields in stimulated cortex (Bikson et al., 2016; Woods et al., 2016). Ample evidence suggests that stimulation modulates cortical activity by making neurons in stimulated cortex slightly more or less likely to fire an action potential (Jamil et al., 2017; Nitsche et al., 2008; Stagg & Nitsche, 2011). This modulation of neuronal excitability, in turn, affects cognitive processes such as memory (Kronberg et al., 2017; Kronberg et al., 2020; Woods et al., 2016). Past work has shown that memory can be improved under active versus sham stimulation. For example, in one investigation, participants studied face‐name pairs under either active or sham stimulation (Leshikar et al., 2017). At test, participants were shown a face, and asked to retrieve the name associated with that face. Results showed improved memory under active versus sham stimulation, consistent with the idea that tDCS can improve memory. Only recently have researchers started investigating the influence of tDCS on the self‐reference effect in memory. Interestingly, these past studies show tDCS does not increase the magnitude of the self‐reference effect in memory (Mainz et al., 2020; Martin et al., 2019). It is worth noting however, that this past work has focused on item memory (e.g., Mainz et al., 2020), and thus it is less known the extent that self‐reference context memory effects might be influenced under active versus sham stimulation. Thus, in this investigation we examine the influence of tDCS on self‐reference context memory effects.

When devising tDCS investigations of memory, it is important to choose stimulation sites that are known to support memory effects of interest (Nitsche et al., 2008). Because we are interested in self‐reference context memory effects, it is critical to place stimulating electrodes over a cortical site known to support this type of memory. In our past work, we have identified cortical areas associated with self‐reference context memory effects. Specifically, in an fMRI investigation (Leshikar & Duarte, 2014), participants studied common objects (e.g., bottle) superimposed on one of three background scenes (i.e., mountain, beach, desert). In the self‐reference condition, participants judged whether they found the image (e.g., bottle) superimposed on that background (e.g., mountain scene) pleasant. In the other‐reference condition, participants judged whether the Queen of England would find that object‐background pair pleasant. At retrieval (memory test), participants were shown an object and then made an item recognition judgment (is this item old or new?), as well as a context memory judgment (with which background was this object paired?). Results showed a strong self‐reference context memory effect, where memory for the backgrounds was better in the self‐reference compared to the other‐reference condition. Importantly, results also indicated that a region of dorsal medial prefrontal cortex (dmPFC) selectively supported context memory for self‐referentially processed materials. Specifically, dmPFC activity during study, when participants were encoding materials, supported the self‐reference context memory effect (Leshikar & Duarte, 2014), which fits with past work demonstrating that medial prefrontal cortex supports thinking about materials with respect to the self (Moran et al., 2009; Northoff & Bermpohl, 2004; Qin & Northoff, 2011; Schäfer & Frings, 2019; Schneider et al., 2008), as well as associated with self‐reference effects in memory (Craik et al., 1999; Kelley et al., 2002; Macrae et al., 2004; Moran et al., 2006; Northoff et al., 2006; Wong et al., 2017). In the current investigation, we stimulated dmPFC at the time participants studied materials to investigate the extent self‐reference context memory effects are affected by tDCS. We were especially interested in whether the self‐reference context memory effect might be increased under active compared to sham stimulation, consistent with the idea that tDCS can modulate memory.

In this investigation, we examine the self‐reference context memory effect for active versus sham tDCS applied to the dmPFC during study (i.e., encoding). Similar to our prior work (Leshikar & Duarte, 2012, 2014), participants studied objects superimposed on background scenes under both self‐reference and other‐reference conditions. We make two predictions in this investigation. First, we expect to find a self‐reference context memory effect, with better memory for stimuli processed in the self‐reference compared to the other‐reference condition. Such a finding will extend past work showing that self‐referential processing is a powerful mnemonic that improves memory for a variety of episodic details (Rogers et al., 1977; Symons & Johnson, 1997), including context memory (Dulas et al., 2011; Hamami et al., 2011; Serbun et al., 2011). We also expect a self‐reference effect for item memory, consistent with past work (Symons & Johnson, 1997). Second, we predict one of two possible outcomes for tDCS‐induced memory effects. Given that past work shows that tDCS can improve memory (Bjekić et al., 2019; Brasil‐Neto, 2012; Hsu et al., 2015) and that we chose to stimulate a region known to support context memory for self‐referentially processed materials, we predict the magnitude of the self‐reference context memory effect to be larger under active compared to sham stimulation. Alternatively, because limited past work has shown that the self‐reference effect is not enhanced by tDCS (Mainz et al., 2020; Martin et al., 2019), it is possible that stimulation will not affect the self‐reference context memory effect. Either outcome (enhanced self‐reference context memory effect; no effect of tDCS on memory) will extend understanding of the effects of tDCS on context memory for self‐referentially processed information.

## 1 METHODS

### 1.1 Participants

A total of 28 participants (22.8: Mean Age, 3.9: SD Age, 18 females) were recruited and completed experimental procedures. An a priori power analysis based on effects from prior work using this same task (Leshikar & Duarte, 2014) showed that 14 participants per group were needed to attain 0.80 power with an alpha of 0.05. All participants were healthy, right‐handed adults. Participants were screened for contraindications for tDCS including metal implants in their body, personal or familial history of epilepsy, psychoactive drug use, scalp abrasions, skull fractures, brain injury, and brain surgery. All participants were recruited from the Chicago area and were paid for their participation.

### 1.2 Materials

Stimuli consisted of 356 common objects (bottle, hat, ring, dog, chair, etc.) and three background scenes (mountain, beach, desert), as used before (Leshikar & Duarte, 2014). During encoding, 264 objects were superimposed on one of the three background scenes and 92 objects were shown as novel lures at test. Across participants, stimuli were counterbalanced so that objects were paired with each of the different background scenes and appeared in both the self‐reference and other‐reference conditions. Further, objects were counterbalanced to be shown either as items seen at encoding (study) or as novel items at retrieval (test).

### 1.3 Procedure overview

The experiment was conducted in a single session. The experiment consisted of two phases: an encoding (study) phase, and a retrieval (test) phase. In the experiment, participants were first prepared for stimulation, which was following by training that included instructions on how to complete the encoding and recognition phases of the experiment. Then participants completed encoding during which tDCS was applied. Following encoding, participants who completed recognition (not stimulated), were debriefed and dismissed.

### 1.4 tDCS

We applied stimulation to participants using Activatek ActivaDose II Controllers using two square sponge electrodes, as done before (Clark et al., 2012). Sponges measured 3.3 cm per side. Stimulation locations were first prepped using alcohol swabs. We placed the anodal (positive) electrode over Fz according to the International 10–20 system, and the cathodal (negative) electrode on the right upper arm (extracephalic location). Electrodes were held in place using medical banding. We chose Fz because this location approximates the position of a dmPFC region known to support self‐reference context memory effects in past fMRI work (Leshikar & Duarte, 2014). Participants received either active (1.5 mA) or sham (0.1 mA) stimulation. Past work has shown that 0.1 mA stimulation mimics sensations attributable to stimulation (Leach et al., 2019), without affecting cortical excitability (Miranda et al., 2009). For both active and sham stimulation, current was ramped up to full dosage (for each respective stimulation condition) over 30 s, and then ramped down over 30 s at the end of stimulation. Stimulation lasted exactly 30 min for all participants. Stimulation was double‐blinded such that neither the participant nor the experimenter knew the stimulation condition (active or sham). To achieve double blinding, two stimulators were connected to a blinding box. One stimulator was set to active stimulation and the other to sham stimulation. The blinding box then sent stimulation from only one of the stimulators to the participant. To help confirm that our montage stimulated our primary region of interest, the dmPFC, we ran a computational model of the electric field intensity using our stimulation protocols. Results of the modeling suggest our montage‐stimulated dmPFC (see **1**), including a region known to support self‐reference context memory effects (Leshikar & Duarte, 2014).

**FIGURE 1:**
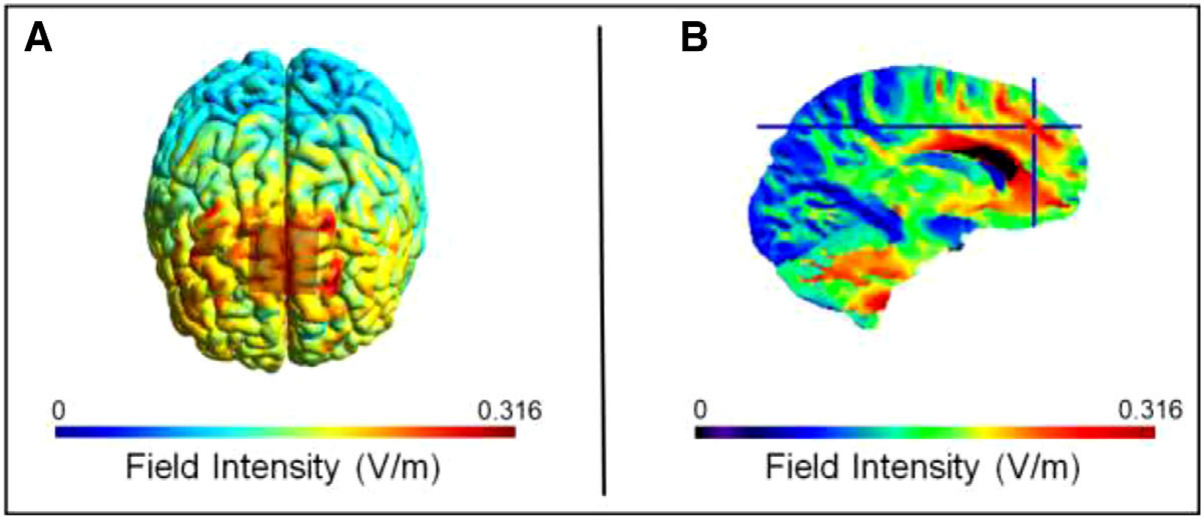
Depiction of computational model of stimulation‐induced field intensity in cortex using our stimulation montage. In (a), results of the model showing field intensity on the cortical surface, as well as the overlay of the anode electrode placement over cortex. In (b), results of the model show the field intensity in sagittal slice depicting the dmPFC. The crosshairs show a region of dmPFC known to support self‐reference context memory effects in past fMRI work (Leshikar & Duarte, 2014)

### 1.5 Procedure

After signing the informed consent, participants were prepared for stimulation (placing electrodes, etc.). Before stimulation started, participants were given training and practice trials on how to complete the encoding (study) phase, and the retrieval (test) phase of the experiment. As part of the training, participants completed 18 practice encoding trials and 11 practice retrieval trials. Participants were given an opportunity to ask clarifying questions about the instructions for the encoding and retrieval phases of the experiment.

We started stimulation after training but before encoding. Participants waited silently for 2 min to habituate to tDCS‐induced sensations. After 2 min, participants completed a sensation questionnaire to rate the amount of itching, burning, tingling, and fatigue, respectively, they currently were experiencing on a 1 (very mild) to 10 (extremely high) scale. If any participant reported a 7 or higher on any rating, the experiment was discontinued (no participant did so). The sensation questionnaire was administered four more times between encoding blocks (approximately every 4 min).

Four minutes after stimulation was initiated, participants started the encoding phase of the experiment. Participants completed a total of 264 encoding trials over four encoding blocks (66 encoding trials per block). At encoding, participants were shown an object (e.g., bottle) superimposed on one of three background scenes (e.g., mountain; see **2**). Participants studied items in two different encoding tasks. In the self‐reference task, participants judged whether they found the object‐scene pairing pleasant (yes/no). This is a type of self‐reference task used in prior studies (Dulas et al., 2011; Leshikar & Duarte, 2014). In the other‐referent task, participants judged whether they think Queen Elizabeth II would find the object‐scene pairing pleasant (yes/no), as done before (Leshikar & Duarte, 2012, 2014). Participants made all yes/no decisions on a keyboard using the index and middle finger of their right hand (v = yes, b = no). On each encoding trial participants viewed the object‐scene pair and had 3500 ms to rate each pair (“would you/the Queen like this pairing?”), which was followed by a 250 ms fixation between trials.

**FIGURE 2:**
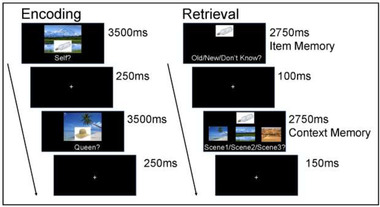
Trial schematic for the encoding (study) and retrieval (test) phases of the experiment

**FIGURE 3:**
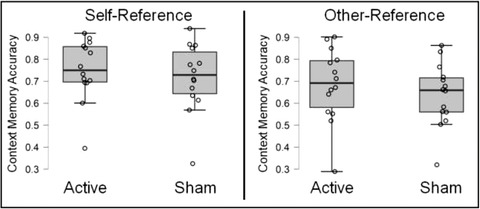
Context memory accuracy is shown for the self‐ and other‐reference conditions, as a function of stimulation (active, sham). Results showed a self‐reference context memory effect driven by better memory for information processed in the self‐compared to the other‐reference condition, under both active and sham stimulation

After stimulated encoding, participant completed the retrieval phase of the experiment (not stimulated). Participants completed 356 retrieval trials which consisted of both old (264 items seen at encoding) as well as novel objects (92 new objects not seen at encoding). On each retrieval trial, participants made two decisions: an item memory and a context memory decision. On each trial, participants were shown a single object (e.g., bottle) and were given 2750 ms to decide whether the object was old, new, or whether they did not know, which served as our item memory decision. With the same object (e.g., bottle) still on screen, participants were then shown all three background scenes and given 2750 ms to decide which scene was paired with the object at encoding. This served as our context memory decision. Trials were separated by a 150 ms fixation. For both retrieval phase decisions (item, context), we included a “do not know” response option to reduce the influence of guessing in this phase of the experiment, as has been done before (Duarte et al., 2008; Gottlieb et al., 2010; Morcom et al., 2007; Smith et al., 2004).

## 2 RESULTS

In this section, we report encoding phase as well as retrieval phase responses. At encoding, participants reported “yes” to the pleasantness question on 45% of the trials in the self‐reference condition, and 37% of the trials in the other‐reference condition, which is consistent with prior work (Leshikar & Duarte, 2012, 2014). For retrieval phase data, item and context memory responses for both old and new items are presented in **1** as a function of encoding task and stimulation condition (active, sham). First, for item memory, we calculated a corrected measure of memory by subtracting the false alarm rate from the item hit rate for materials studied in the self‐reference and other‐reference condition, respectively. Second, for context memory, we calculated estimates of memory accuracy for items in the self‐reference and other‐reference condition, respectively, by calculating the proportion of trials where context memory was correct out of the proportion of trials where they made context memory attributions (context correct/[context correct + context incorrect]), as done before (Bayen et al., 1996; Leshikar & Gutchess, 2015; Leshikar et al., 2014; McCurdy et al., 2017, 2019, 2021; McCurdy, Sklenar, et al., 2020). For both item and context memory, we performed 2 (encoding condition: self‐reference, other‐reference) × 2 (stimulation condition: active, sham) analyses of variance. For all analyses, alpha was set at 0.05.

**TABLE 1:** Retrieval phase responses for item memory and context memory decisions presented as a function of encoding condition (self‐reference, other‐reference) and stimulation condition (active, sham) as well as responses to novel items

Memory results							
Active stimulation							
Item memory				Context memory			
Task	Old	New	Don't know	Task	Context correct	Context incorrect	Don't know
Self‐reference	0.79 (0.18)	0.15 (0.11)	0.06 (0.07)	Self‐reference	0.47 (0.17)	0.16 (0.07)	0.37 (0.16)
Other‐reference	0.78 (0.20)	0.15 (0.12)	0.07 (0.09)	Other‐reference	0.39 (0.14)	0.18 (0.09)	0.43 (0.14)
New	0.07 (0.06)	0.85 (0.14)	0.08 (0.08)				

Sham stimulation							
Item memory				Context memory			
Task	Old	New	Don't know	Task	Context correct	Context incorrect	Don't know
Self‐reference	0.86 (0.07)	0.10 (0.07)	0.04 (0.04)	Self‐reference	0.47 (0.14)	0.21 (0.14)	0.32 (0.17)
Other‐reference	0.83 (0.09)	0.12 (0.09)	0.05 (0.05)	Other‐reference	0.43 (0.15)	0.21 (0.13)	0.36 (0.18)
New	0.07 (0.10)	0.87 (0.13)	0.06 (0.09)				

For item memory, analysis of variance results showed a marginal effect of encoding condition, *F*(1, 26) = 2.89, *p* = 0.10, *η*_p_^2^ = 0.10, with numerically higher item memory for the self‐reference (*M* = 0.76, SE = 0.03) compared to the other‐reference condition (*M* = 0.74, SE = 0.04). The stimulation effect was not significant, *F*(1, 26) = 0.56, *p* = 0.46, *η*_p_^2^ = 0.02, nor was the interaction *F*(1, 26) = 0.86, *p* = 0.36, *η*_p_^2^ = 0.03.^1^

For our primary analysis, we examined context memory effects (See **Figure** **3**). Results revealed a significant effect of encoding condition, *F*(1, 26) = 10.71, *p* = 0.003, *η*_p_^2^ = 0.29, which was driven by better memory in the self‐reference (*M* = 0.72, SE = 0.03) compared to the other‐reference condition (*M* = 0.68, SE = 0.03). This is consistent with past work showing self‐reference context memory effects (Dulas et al., 2011; Hamami et al., 2011; Serbun et al., 2011). The main effect of stimulation was not significant *F*(1, 26) = 0.51, *p* = 0.48, *η*_p_^2^ = 0.02, and the encoding condition by stimulation interaction was not significant, *F*(1, 26) = 0.32, *p* = 0.57, *η*_p_^2^ = 0.01.^2^ Because frequentist statistics (e.g., traditional analysis of variance) cannot be used to interpret null findings, we used a Bayesian approach to understand whether the null results (no main effect of stimulation or stimulation by condition interaction) are meaningful. To do so, we performed a Bayesian analysis of variance. Results of this analysis showed a Bayes Factor (BF01) of 19.27 for stimulation effects on context memory, which suggests that the null hypothesis (no effect of stimulation on context memory) is 19 times more likely than the alternative hypothesis (that stimulation affects context memory), which is substantial evidence in favor of the null hypothesis (Jeffreys, 1998). Further, results showed a Bayes Factor (BF01) of 3.68 for the interaction between condition and stimulation, suggesting the null was three times more likely than the alternative hypothesis (that stimulation would interact with encoding condition). Past work suggests that a Bayes factor greater than 3 is modest evidence in favor of the null (Jeffreys, 1998). Overall, Bayesian analysis results support the idea that tDCS had no effect on context memory, and did not increase the magnitude of the self‐reference context memory effect.^3^

## 3 DISCUSSION

In this investigation, we examined the influence of tDCS applied at encoding over the dmPFC on the self‐reference context memory effect. We have two primary findings in this report. First, we found a self‐reference context memory effect, where memory was better in the self‐reference compared to the other‐reference condition. Such a finding is consistent with the idea that processing information in relation to the self has a strong influence on memory (Symons & Johnson, 1997). Second, tDCS did not have an influence on either item or context memory in these data. Such a finding is in line with past work that stimulation of the dmPFC does not increase the magnitude of the self‐reference effect in memory (Mainz et al., 2020; Martin et al., 2019). Together these data suggest self‐referencing has a powerful effect on memory, but that tDCS does not enhance the memory benefits attributable to self‐referential processing, at least under the stimulation parameters we used in this investigation.

In this experiment, we found a self‐reference context memory effect. Such a finding is consistent with emerging work that self‐referencing improves memory for many types of contextual, episodic details. Past work has identified several potential memory mechanisms underlying the self‐reference effect, such as enhanced elaborative processing of studied items (Klein & Kihlstrom, 1986; Kuiper & Rogers, 1979; Rogers et al., 1977). Interestingly, a recent theoretical perspective suggests that self‐referencing induces enhanced integration of various episodic details into a retrievable memory store (Humphreys & Sui, 2016; Sui, 2016; Sui & Humphreys, 2015). Specifically, this perspective suggests that self‐referential processing improves memory by binding various episodic details, in service of making self‐relevant information especially accessible in memory. According to this perspective, such improved memory for episodic details leads to improved source memory (memory for contexts in which an item was encountered), as well as enhanced memory for perceptual details associated with an episode. Our self‐reference context memory finding is consistent with this theoretical account, where participants were better able to remember the background scene with which an item was paired. Although we found a self‐reference context memory effect, we did not find a self‐reference effect for item memory. One possible reason that we did not find a self‐reference item memory effect is because performance on this measure was approaching ceiling, and thus there may have been less room for item memory to improve under self‐referential processing conditions. Although we expected to see a self‐reference item memory effect, it is worth noting that past investigations have not always found item memory self‐reference effects (Bower & Gilligan, 1979; Kuiper & Rogers, 1979), thus the present findings have some precedent. For example, in our prior work, we found strong self‐reference effects as measured by context memory, but not item memory using a similar memory procedure (Leshikar & Duarte, 2014). Future work may be necessary to understand the experimental conditions under which different self‐reference memory effects may emerge for item versus context memory.

In this experiment we examined the influence of tDCS applied over dmPFC at encoding on the self‐reference context memory effect. We chose to place our stimulating electrode over the dmPFC because past work has shown this region is involved in selectively supporting self‐reference context memory effects (Leshikar & Duarte, 2014). We found no evidence, however, that tDCS influenced context memory in this investigation. The fact that we did not find a boost to memory for self‐referentially processed items or their context is consistent with the few investigations that have measured tDCS effects on self‐referential processing (Mainz et al., 2020; Martin et al., 2019). Specifically, this past work has shown that tDCS does not increase the magnitude of the self‐reference effect in memory. Thus, our findings, taken in conjunction with this limited past work, may suggest that tDCS does not additively build upon the memory benefits induced by self‐referential encoding for items or contexts. Interestingly, much of the past work showing tDCS‐induced memory improvement in non‐self‐reference experiments have targeted more lateral regions of the prefrontal cortex, especially the dorsolateral prefrontal cortex (Javadi & Walsh, 2012; Leshikar et al., 2017; Manenti et al., 2017; Zwissler et al., 2014), which follows the general trend in tDCS work where lateral prefrontal cortex is the most common stimulation target (Ghobadi‐Azbari et al., 2021). In contrast, studies investigating self‐reference effects have stimulated medial (midline) prefrontal regions (Mainz et al., 2020; Martin et al., 2019). This difference in stimulation location (medial versus lateral prefrontal cortex) is potentially important, because past work on tDCS effects on neuronal excitability suggests that the orientation of cortical neurons relative to the stimulating electrodes (e.g., how the layers of neurons in cortex are oriented relative to the stimulating electrodes) can affect neuronal excitability (Bikson et al., 2004). Thus, one possible reason we, and others, have not found tDCS effects on memory for self‐referential processing, may be related to the orientation of neurons in midline regions relative to the stimulating electrode, which may make stimulation of these regions (dmPFC) less effective in modulating memory for self‐referentially processed materials. Although our computational model of stimulation field intensity suggests we stimulated our region of interest, future work could try to target the dmPFC using a different approach such as placing electrodes on the left and right lateral frontal cortex to better target neurons in the medial wall of the dmPFC.

Although we found that stimulation of the dmPFC at encoding had no effect on memory, the results of the current investigation have several implications for use of tDCS on the self‐reference effect in memory. First, in the procedures we used participants completed retrieval immediately following encoding. Past work has shown that the effects of stimulation on cortical excitability persist for a period of minutes to hours after stimulation ceases (Reinhart et al., 2017). Thus participants in our investigation were likely still experiencing carry‐over stimulation effects on cortical function at the time of retrieval. This is relevant because this implies that stimulation of the dmPFC at encoding, as well as possible stimulation carry‐over effects at the time of retrieval do not strongly affect memory for self‐referentially processed materials. Future work might try to apply tDCS to the dmPFC at retrieval to confirm that stimulation during the memory test does not strongly modulate self‐reference context memory effects. Second, past tDCS work on memory shows that stimulation of cortex, such as areas of the prefrontal cortex, can induce heightened functional connectivity with other regions of the brain known to support memory, such as the hippocampus (Antonenko et al., 2019). Although speculative, the fact that we did not observe improved self‐reference context memory effects under active compared to sham stimulation could imply that stimulation of the dmPFC is not sufficient to induce functional connectivity changes between memory‐related regions (dmPFC; hippocampus) that lead to enhanced memory. Future work might be necessary to understand whether other stimulation parameters could affect the self‐reference effect in memory, which is aligned with work in other domains examining stimulation parameters that have the largest effects on different cognitive processes (Agboada et al., 2019; Dedoncker et al., 2016; Friehs, Frings, et al., 2021; To et al., 2016).

Interestingly, research suggests that both self‐referential memory effects and tDCS‐induced memory effects may be partially due to *enhanced recollection* processes. Recollection is the ability to retrieve specific episodic details associated with past experiences. Prior work on the self‐reference effect suggests the boost in memory from self‐referential processing may partially derive from *enhanced recollection* of specific episodic detail (Conway & Dewhurst, 1995). For example, in one investigation participants studied words in either a self‐reference or semantic processing control condition (Leshikar et al., 2015). Participants then made “remember” judgments (indicating they could recollect specific episodic details) or familiarity judgments (indicating they knew the word was old but without remembering any specific episodic details) about stimuli. Results showed that self‐referential processing was associated with a higher proportion of “remember” responses, consistent with the idea that self‐referencing enhances recollection relative to control. Turning back to the present investigation, our finding of a self‐reference context memory effect is aligned with the idea that self‐referencing improves the ability to recollect specific episodic details, such as with which background an object was paired. Similarly, memory‐related work in the tDCS literature suggests that stimulation also operates to *enhance recollection* processes that lead to stimulation‐induced memory improvement (Gray et al., 2015; Leshikar et al., 2017). Because past work on the self‐reference effect as well as tDCS work implicates enhanced recollection as a possible mechanism through which memory improves, it was plausible to predict additive effects of tDCS and self‐referencing on memory for contextual details, but that is not what we found. Instead, we found that tDCS had no additional effects on memory, over and above that induced by self‐referential processing. It may be that enhanced recollection due to self‐referencing is sufficiently robust that there are no additive boosts to recollection processes that derive from stimulation. This is consistent with work in other memory domains showing that separate factors known to improve memory (such as encoding strategies; stimulation effects) in isolation may not be additive to yield even larger memory improvements when such separate factors are combined (Spataro et al., 2021). Understanding ways to improve memory is an important pursuit (Bjork & Benjamin, 2011; Frankenstein et al., 2020; Giannakopoulos et al., in press; Jennings et al., 2005; McCurdy, Viechtbauer, et al., 2020; Meyers et al., 2020; sklenar et al., in press; Villaseñor et al., 2021; Yonelinas, 2002), and the findings of this experiment are in line with that experimental goal.

In this experiment, we found a self‐reference context memory effect driven by improved context memory for self‐referentially compared to other‐referentially processed materials, and further, that tDCS did not affect memory. Although this work advances understanding of stimulation effects on memory, including the self‐reference effect, there are two limitations of the investigation worth describing. First, given that item memory performance was approaching ceiling, and that we did not find a self‐reference item memory advantage, it is possible that our memory test for items may not have been sufficiently challenging to truly find self‐reference item memory effects. Future work might use an item memory task to pull performance from ceiling to better understand the possible influence of tDCS on item memory self‐reference effects. Second, in our experimental procedures, participants were judging whether object‐scene pairings were pleasant as our self‐reference task. Although other investigations have used similar tasks to induce self‐referential processing (Dulas et al., 2011; Johnson et al., 2005; Zysset et al., 2002), it is possible that the self‐reference effects we observed in this investigation were smaller in magnitude compared to what they would be in a task that more directly induced self‐referential processing (e.g., judging whether words describe the self). Thus, future work might use a more traditional self‐reference task, such as judging whether information is descriptive of the self to further understand the influence of tDCS on the self‐reference effect in memory.

In this investigation, we found a self‐reference context memory effect driven by enhanced memory for materials processed in reference to the self‐compared to an other‐reference condition. Such a finding adds to the literature that self‐referencing has a strong influence on memory, including memory for contextual details. We found no evidence, however, that tDCS affected memory, which suggests limits to the conditions under which stimulation may modulate memory. Overall, these data advance understanding on how self‐referencing and tDCS affect memory.

## 4 FUNDING INFORMATION

Publication of this work was supported by the Research Open Access Article Publishing Fund at the University of Illinois at Chicago.

## Footnotes

1 In addition to our main analysis of item memory, we also calculated signal detection measure *d’* which takes into account both hits and false alarm rates (Snodgrass & Corwin, 1988). We analyzed *d’* estimates of item memory using a 2 (encoding condition: self‐reference, other‐reference) × 2 (stimulation condition: active, sham) analysis of variance. Results fully replicated our primary context memory analysis. Specifically, we no effect of encoding condition, stimulation condition, or interaction, *Fs* < 4.01, *ps* > .06, *η*_p_^2^ < 0.13.

2 In the retrieval procedures we used, it is possible that participants could have inaccurate item memory for a trial, but then successfully retrieve the context for that same item. Because such trial‐types would be a challenge to interpret, we further analyze our context memory data conditionalized on accurate item recognition. Specifically, we examined context memory accuracy only for those trials associated with accurate item memory. We examined this conditionalized context memory measure in a 2 (encoding condition: self‐reference, other‐reference) × 2 (stimulation condition: active, sham) analysis of variance. Results fully replicated our primary context memory findings: we found an effect of condition, *F*(1, 26) = 19.03, *p* < .001, *η*_p_^2^ = 0.42, where memory was better for the self‐ compared to the other‐reference condition. The effect of stimulation condition, *F*(1, 26) = 0.20, *p* = 0.66, *η*_p_^2^ < .01, and the stimulation by condition interaction, *F*(1, 26) = 0.11, *p* = 0.74, *η*_p_^2^ < .01, were not significant.

3 To further analyze our context memory data, we calculated another measure of context memory called Psr. Psr is an estimate of context memory that removes the possible influence of guessing from performance (Duverne et al., 2008). Psr is derived as follows: Psr = (p(context correct) – 0.5 × (1 – p(“don't know”)))/(1 – (0.5 × (1 – p(“don't know”)))). We analyzed Psr estimates using a 2 (encoding condition: self‐reference, other‐reference) × 2 (stimulation condition: active, sham) analysis of variance. Results again replicated our primary context memory analysis. Specifically, we found an effect of encoding condition, *F*(1, 26) = 14.93, *p* = 0.001, *η*_p_^2^ = 0.37, but no effect of stimulation condition, *F*(1, 26) = 0.01, *p* = 0.93, *η*_p_^2^ < .01, or interaction, *F*(1, 26) = 1.11, *p* = 0.30, *η*_p_^2^ = 0.04. This analysis further confirms our self‐reference context memory findings.
